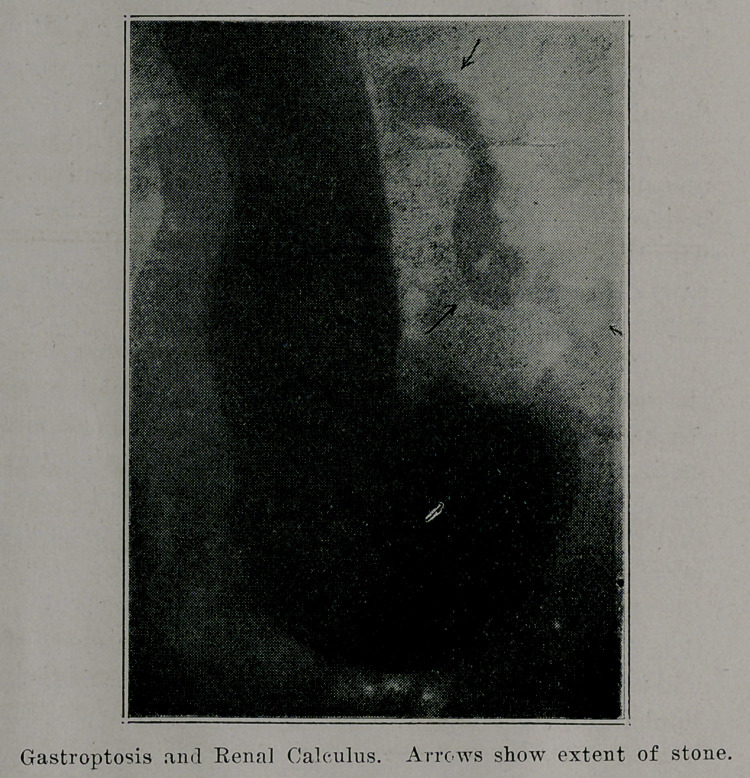# A Clinical Paradox

**Published:** 1916-03

**Authors:** George M. Niles

**Affiliations:** Atlanta, Ga.


### A CLINICAL PARADOX.

By George M. Niles M. D., Atlanta, Ga.

This brief clinical narrative illustrates nature's tolerance for markedly abnormal conditions in the abdomen and kidney.

Mrs. C. P., referred to me by Dr. L. P. Hammond of Rome, Ga., came for a clearing up of obscure pathology in the right iliac region, concerning which there had been some divergence of medical opinion.

The accompanying roentgenogram shows a large, branched stone in the pelvis of the right kidney, while her stomach, much elongated and ptosed, extends well down into the pelvis. As we expected, other roentgenograms disclosed extreme ptosis of all the intestines, especially the transverse colon.

Gastroptosis and Renal Calculus. Arrows show extent of stone.

Strange to relate, she gave a history of no renal colic, of no indigestion, nor constipation. Apart from rather frequent and imperative urination, and occasional presence of pus in the urine, she reported herself a normal and exceptionally active woman.

Though of slender physique, she was energetic in body and vivacious in mind—in fact, she presented the antithesis of the clinical picture which might be anticipated from her manifold infirmities, both pathologic and "geographic."

## Figures and Tables

**Figure f1:**